# Neoadjuvant Pembrolizumab Associated with Chemotherapy in Early Triple-Negative Breast Cancer Patients: Real-World Data from a French Single-Center Experience

**DOI:** 10.3390/cancers18030358

**Published:** 2026-01-23

**Authors:** Ichrak Ben Abdallah, Severine Guiu, Xavier Quantin, William Jacot, Philine Witkowski

**Affiliations:** 1 Institut de Recherche en Cancérologie de Montpellier, 34298 Montpellier, France; 2 IRCM, University Montpellier, ICM, INSERM, 34298 Montpellier, France

**Keywords:** pembrolizumab, triple-negative breast cancer, toxicity

## Abstract

**Introduction:** The addition of immunotherapy to neoadjuvant treatment for early triple-negative breast cancer (TNBC) has been adopted in clinical practice in France since March 2022, with little real-world data published on the topic. The aim of this study was to evaluate real-world data on treatment feasibility, efficacy, and related toxicities, with a specific focus on immune-related adverse events (irAEs). **Methods:** We conducted a retrospective analysis of patients who completed at least the neoadjuvant sequence of pembrolizumab combined with chemotherapy for early-stage TNBC at Montpellier Cancer Institute from April 2022 to July 2024. Adverse events were graded according to the Common Terminology Criteria for Adverse Events (CTCAE) v5.0. The pathological complete response (pCR) was defined as the absence of residual invasive disease in the breast and axillary lymph nodes (ypT0/Tis ypN0). **Results:** We reviewed data from 92 patient records. The median age at diagnosis was 50 years (range: 27–76). The history of autoimmune disease was noted in 3.2% of patients. Grade 3–4 irAEs were observed in 20% of patients and included hepatitis (8.6%), colitis (3.3%), skin toxicity (2.1%), myocarditis (2%), arthralgia (1%), autoimmune hemolytic anemia (1%), hypothyroidism (1%), and adrenal insufficiency (1%). No treatment-related deaths were reported. Immunotherapy was discontinued due to irAEs in 29.3% of patients in the study population. The pCR rate was 61,1%, with no significant association between the number of neoadjuvant pembrolizumab cycles and the pCR rate (*p* = 0.7). Patients experiencing grade 3–4 irAEs had a pCR rate of 80%, compared to 56.7% in those without such toxicities (*p* = 0.079). Initial positivity of antinuclear antibodies (ANA) was not associated with an increased incidence of irAEs. **Conclusions:** The immune-related adverse events and efficacy data observed in our cohort were broadly comparable to those reported in the KEYNOTE-522 trial, with no treatment-related deaths. Patients with grade 3–4 irAEs tended to have higher pCR rates.

## Simple Summary

This retrospective study assessed the real-world feasibility, safety, and efficacy of neoadjuvant pembrolizumab plus chemotherapy for early triple-negative breast cancer (TNBC) at Montpellier Cancer Institute (2022–2024). Ninety-two patients were included (median age 50). Grade 3–4 immune-related adverse events (irAEs) occurred in 20% of patients, most commonly hepatitis, colitis, skin toxicity, myocarditis, and rare hematologic or endocrine events; no treatment-related deaths were reported. Discontinuation of immunotherapy because of irAEs was reported in 29.3% of patients. The pathological complete response (pCR) rate was 61.1%, with no association between pCR and the number of pembrolizumab cycles. Patients experiencing grade 3–4 irAEs showed a trend toward higher pCR (80% vs. 56.7%). Baseline antinuclear antibodies were not linked to increased toxicity. Overall, real-world toxicity and efficacy were consistent with KEYNOTE-522 findings.

## 1. Introduction

Breast cancer is diagnosed at a localized stage in approximately 60% of cases in France [1]. Triple-negative breast cancers (TNBC) account for around 15% of all breast cancers [2].

Beyond the absence of hormone receptors and HER2 expression, TNBC is distinguished by a high degree of genomic instability, frequent DNA damage–repair deficiencies, and high levels of tumor-infiltrating lymphocytes (TILs), making it a particularly immunogenic breast cancer subtype [3,4]. These features have provided a strong biological rationale for the development of immune checkpoint inhibitors (ICIs) in both metastatic and early-stage settings [5].

ICIs were initially validated in metastatic or unresectable locally advanced TNBC. In 2021, pembrolizumab received marketing authorization for metastatic first-line use in combination with chemotherapy based on the results of the KEYNOTE-355 trial [6].

In the neoadjuvant setting, the KEYNOTE-522 study [7] changed the standard of care by demonstrating a benefit from the addition of pembrolizumab (anti-PD-1) to chemotherapy. In France, access to this regimen was initially granted through an early access authorization in March 2022 [8], with official reimbursement following in November 2024 [9].

Given this treatment escalation integrating immunotherapy with chemotherapy in a neoadjuvant setting, the main objective of this study was to assess the tolerance of this regimen, with a focus on immune-related toxicities and their incidence, management, and impact on treatment course.

## 2. Materials and Methods

### 2.1. Study Design and Setting

This is a retrospective descriptive study involving patients treated at the Montpellier Cancer Institute for localized TNBC according to the KEYNOTE-522 regimen [7]. Eligibility required having received neoadjuvant pembrolizumab-based therapy followed by surgery (except in cases where surgery was not performed due to disease progression).

### 2.2. Data Collection and Analysis

The data collected included demographic characteristics, clinical and biological features of the disease, and treatment details. Adverse events were retrospectively extracted from electronic medical records, including outpatient oncology visit reports, hospitalization summaries, laboratory results, imaging reports, pathology findings, and multidisciplinary meetings. Toxicities were graded according to the Common Terminology Criteria for Adverse Events (CTCAE) v5.0. [10].

Descriptive statistics were used to summarize patient characteristics, treatment exposure, and adverse events, and were reported as absolute numbers and percentages for categorical variables, and as medians with ranges for continuous variables. Comparisons between categorical variables were performed using the chi-square test or Fisher’s exact test when expected cell counts were small. All statistical analyses were performed using SPSS software (version 26; IBM Corp., Armonk, NY, USA), and a *p*-value < 0.05 was considered statistically significant. Given the retrospective design and the limited sample size, statistical analyses were primarily descriptive. No multivariable modeling was performed. When Fisher’s exact test was used, a one-sided analysis was performed based on an a priori directional hypothesis informed by previous reports.

## 3. Results

### 3.1. Patient Characteristics and Pre-Treatment Assessment

A total of 92 patients were included in the study. The epidemiological characteristics are presented in Table 1. The median age at diagnosis was 50 years (range: 27–76), with 24% of patients under 40 years and 3.2% over 70 years. At baseline, 3 patients had pre-existing autoimmune disorders: 1 with rheumatoid arthritis, 1 with eczema, and 1 with Hashimoto’s thyroiditis. Pre-treatment biological data, including serum autoantibodies, are summarized in Table 2.

### 3.2. Overall Treatment Tolerance

In the neoadjuvant phase, 68 patients completed all 8 planned neoadjuvant pembrolizumab cycles (74%). Only 43% of patients were able to complete all 12 cycles of paclitaxel–carboplatin. The median number of neoadjuvant paclitaxel–carboplatin cycles received was 11. Treatment interruptions or delays due to clinical and/or laboratory toxicity during this regimen were observed in 67% of patients. During the anthracyclines sequence, 79.3% of patients completed all 4 planned cycles.

In the adjuvant phase, patients who received pembrolizumab accounted for 65% of the cohort. The reasons for omission of adjuvant pembrolizumab in routine practice were investigated and are detailed in Table 3 (N = 32).

The main toxicities observed during treatment are presented in Table 4. It is noteworthy that immunotherapy was discontinued due to potentially immune-related toxicity in 29.3% of cases. No deaths related to immunotherapy or chemotherapy were reported during the treatment period. Grade 3–4 irAEs affected 20% of the study population and comprised the following: hepatitis (8.6%), colitis (3.3%), skin toxicities (2.1%), myocarditis (2%), arthralgias (1%), autoimmune hemolytic anemia (1%), hypothyroidism (1%), and adrenal insufficiency (1%).

No association was found between the occurrence of grade 3–4 toxicities and baseline antinuclear antibody status (positive or negative).

### 3.3. Liver Toxicity

Thirty-six percent (36%) of patients in the study experienced liver function test abnormalities during treatment, with 31.5% occurring during the neoadjuvant phase. These were grade 1–2 in 29.3% of cases, grade 3–4 in 8.6%, and grade 4 in 2.2%, according to the CTCAE v5.0 classification. A liver biopsy was deemed necessary in 9.4% of the cases, confirming immune-related hepatitis. The biological characteristics, severity grading, management, and clinical outcomes are detailed in Supplementary Table S1.

It is worth noting that no significant association was found between the presence of antinuclear antibodies (ANA) at baseline and the occurrence of liver function test abnormalities (*p* = 0.52). Similarly, there was no association between the grade of hepatic involvement and ANA status (positive or negative) at the time of toxicity (*p* = 0.6). Finally, all patients who were ANA-positive at the time of hepatic toxicity were ANA-negative at baseline.

### 3.4. Cutaneous Toxicity

Cutaneous toxicity (excluding nail disorders and other adnexal abnormalities attributable to chemotherapy) was observed in 32.6% of patients during the study. Table 5 summarizes the various cutaneous manifestations observed.

### 3.5. Endocrine Toxicity

New-onset thyroid dysfunction occurred in 27% of patients. Among them, 14% were identified during an initial hyperthyroid phase, while the remaining patients were diagnosed directly at the hypothyroid stage.

New-onset adrenal insufficiency was reported in 8.6% of cases. These toxicities are primarily attributed to immunotherapy due to their underlying mechanisms. Table 6 summarizes data regarding the timing of onset, severity grade, and associated immunological features.

### 3.6. Colitis

Diarrhea was reported in 44.6% of patients, occurring predominantly (95.2%) during the neoadjuvant phase. It was grade 1–2 in 92.7% of cases. All patients who experienced grade 3–4 diarrhea (N = 3, i.e., 3% of the cohort) underwent gastrointestinal endoscopy with biopsies.

To provide a clearer understanding of these severe events, the clinical, endoscopic, and histological characteristics of the three affected patients are detailed in Supplementary Table S2.

### 3.7. Cardiac Toxicity

An elevated troponin level was observed in 16.3% of patients (N = 15). A formal diagnosis of myocarditis was confirmed in only two patients (2%), referred to as Patient A and Patient B later in the text.

Notably, elevated troponin levels were detected in nine patients (9.7% of the total cohort) during the pre-treatment assessment for the adjuvant phase, within a postoperative context. Among them, one (Patient A) underwent a cardiac MRI that revealed features consistent with myopericarditis, highly suggestive of an immune-related etiology, according to the Lake Louise criteria [11]. The eight other patients (8.6%) were not administered pembrolizumab in the adjuvant setting, following the precautionary recommendation of their referring oncologist. Their troponin levels returned to the normal range during follow-up.

As for Patient B, her troponin levels were elevated to 35 pg/mL (compared to <10 pg/mL at baseline) during adjuvant pembrolizumab, despite normal findings on echocardiogram, cardiac MRI, and electrocardiogram (ECG). An endomyocardial biopsy was performed, revealing nonspecific myocarditis with a minimal lymphocytic infiltrate. These results reflected either a pauci-lesional form of immune-mediated myocarditis or a benign postoperative inflammatory reaction. Considering that surgery and myocardial biopsy were separated by a 5-month interval, postoperative context-related myocarditis was unlikely. The immunotoxicity board therefore established the diagnosis of immune-mediated myocarditis.

### 3.8. Pathological Response

A pathological complete response (pCR) was achieved in 61.1% of patients. The distribution of pathological responses according to the Residual Cancer Burden (RCB) score was as follows: RCB score 0 (57.6%), score 1 (7.6%), score 2 (16.3%), score 3 (3.3%), and not assessable (15.2%).

Patients who experienced grade 3–4 immune-related toxicities tended to have a higher pCR rate compared with those without such toxicities (80% vs. 56.8%). A one-sided Fisher’s exact test indicated a trend toward this association (*p* = 0.079), although the difference did not reach conventional statistical significance.

There was no significant association between pCR rate and the number of neoadjuvant pembrolizumab cycles received (*p* = 0.7).

Survival analyses (disease-free survival and overall survival) and the impact of grade 3–4 toxicities on survival remain immature (median follow-up of 22 months) and will be addressed separately after a longer follow-up.

## 4. Discussion

In this study, we evaluated the real-world tolerability of the new neoadjuvant immunochemotherapy regimen (KEYNOTE-522 protocol) in patients with localized TNBC.

In our cohort, the initial phase of the protocol combining pembrolizumab, carboplatin, and paclitaxel (PCP) was marked by numerous treatment delays and cancellations, mainly due to hematological toxicities. Grade 3–4 neutropenia was the most frequent adverse event, occurring in 43.5% of patients, although febrile neutropenia remained rare (2.2%). Conversely, the epirubicin–cyclophosphamide–pembrolizumab (ECP) sequence was associated with a higher incidence of febrile neutropenia (17.4%).

Beyond hematologic events, the most common adverse events during the PCP phase included hypersensitivity reactions, skin rash, peripheral neuropathy, diarrhea, and elevated alanine aminotransferase (ALT) levels.

The most frequent grade 3–4 immune-related adverse events (irAEs) in our cohort were, in decreasing order, hepatitis (8.6%), colitis (3.3%), dermatologic toxicity (2.1%), myocarditis (2%), disabling arthralgia (1%), autoimmune hemolytic anemia (1%), life-threatening hypothyroidism (1%), and adrenal insufficiency (1%). No cases of immune-related pneumonitis were reported. Pembrolizumab was discontinued due to toxicity in 29.3% of patients in our study—a higher rate than that reported in KEYNOTE-522, where 20% of patients discontinued due to adverse events, most commonly pneumonitis (1.9%), diarrhea (1.7%), and elevated transaminases (1%) [12]. However, this discontinuation rate aligns with real-world data from Gustave Roussy, which reported a 35% discontinuation rate in 63 patients [13]. This discrepancy between real-world and clinical trial data may reflect a more preventive approach adopted by oncologists in curative settings for young patients, where the margin for error is minimal—particularly in the neoadjuvant setting, where a severe or prolonged complication could jeopardize subsequent treatment steps, including surgery. In addition, patients treated in routine care may also be less selected than those enrolled in prospective trials, often presenting with comorbidities or baseline fragilities that increase the likelihood of treatment interruption.

Other reasons for pembrolizumab discontinuation, as reported in the Section 3, highlight the uncertainty clinicians face in real-world practice when deciding whether to resume immunotherapy, particularly in patients achieving a pathological complete response (pCR). This uncertainty underscores the need for dedicated clinical trials to better define the role of adjuvant immunotherapy in this context. Ongoing trials such as OptimICE-PCR may help address this question [14].

A notable finding in our series was the detection of elevated troponin levels in 9 patients (9.7% of the cohort) after surgery, during the pre-adjuvant phase treatment assessment. In one of them, cardiac MRI suggested immune-mediated myocardial involvement, which improved without corticosteroids (declined by the patient). In the remaining 8 patients (8.6%), adjuvant pembrolizumab was withheld as a precaution based on their oncologists’ recommendation. Of note, troponin levels returned to a normal range during follow-up. This frequent troponin elevation in the postoperative setting may be consistent with the phenomenon known as myocardial injury after non-cardiac surgery (MINS), which occurs in 10–25% of cases [15]; however, in the absence of systematic myocarditis screening, the distinction between postoperative myocardial injury (MINS) and immune-mediated myocarditis remains hypothesis-generating. Other possible explanations include anthracycline-induced cardiotoxicity administered before surgery or non-ischemic myocardial injury related to systemic inflammation caused by the disease itself.

Among the 92 patients in our cohort, only 2 had a diagnosis of myocarditis (one probably immune-mediated in the postoperative setting as described above, and another with a pauci-lesional myocardial biopsy suggestive of immune-mediated injury during adjuvant therapy), together representing 2.2% of our cohort. Both cases were incidentally detected through mild troponin elevation and had favorable outcomes. This rate is consistent with, or slightly higher than, those in published reports. Indeed, in the KEYNOTE-522 trial, immune-mediated myocarditis occurred in less than 1% of patients (0.4%; three cases among 784 patients) [16]. These observations underscore the importance of biological monitoring, even in asymptomatic patients, although the benefit of systematic myocarditis screening during immunotherapy remains debated [17].

Grades 3–4 hepatic toxicity in our cohort, which accounted for 8.6%, was broadly comparable to that reported in the KEYNOTE-522 trial (5.2%) [18] and to real-world data [13]. Regarding the biochemical phenotype of hepatitis, cholestatic forms were the most common across all grades (42.4%), followed by mixed forms (39%) and cytolytic forms (15%). However, in severe cases (grades 3–4), cytolytic patterns predominated. This distribution aligns with findings in the literature, particularly the multicenter study by Lina Hountondji et al. [19], which reported that PD-1 inhibitor-related hepatitis most frequently presents with a cholestatic or mixed pattern, whereas cytolytic forms are more often associated with anti-CTLA-4 combinations. No patients had positive serum antibodies for autoimmune hepatitis at the time of toxicity. However, antinuclear antibodies (ANA) were detected in 12% of patients at the time of hepatic injury (9% in grade 1 cases and 3% in grade 3 cases), whereas all these patients were ANA-negative at baseline. This phenomenon, suggesting secondary induction of autoimmunity, has been described in the literature. Nilasha et al. reported autoantibody positivity in approximately 18% of patients with ICI-related hepatitis, with no proven prognostic or predictive value [20]. It should be noted that our grading relied on CTCAE v5.0 criteria, as routinely used for immune-related hepatitis. These criteria do not always reflect the true clinical impact of toxicity, as suggested by L. Meunier et al., who recently advocated for alternative hepatology-derived scores (MELD, DILI-IEWG) to better guide therapeutic decisions and assess whether immunotherapy can be safely resumed [21]. We emphasize the critical role of pathological assessment in cases of grade 3–4 liver enzyme elevation during the neoadjuvant phase, given the challenge of distinguishing chemotherapy-induced hepatotoxicity from pembrolizumab-related immune-mediated toxicity.

Regarding cutaneous toxicity, our findings are consistent with KEYNOTE-522 [22], with most cases being maculopapular rash and isolated pruritus, typical of anti-PD-1 therapy [23].

Thyroid dysfunction was observed in 27% of patients—higher than in KEYNOTE-522 (8% hypothyroidism, 3.4% hyperthyroidism) [22]—but in line with other real-world reports (France: 29%, United Kingdom: 26.7%) [13,24]. We found no association between baseline anti-TPO antibody positivity and the development of thyroid dysfunction, contrary to some literature reports [25], possibly due to missing data (half of the patients lacked baseline anti-TPO measurement), which limits interpretation.

Regarding gastrointestinal toxicity, diarrhea was frequent (44.6% vs. 30.4% in KEYNOTE-522) [21], but severe colitis (grade 3) was rare (3% vs. 2.6%). No grade 4 colitis was reported. Among the 3 patients with grade 3 colitis, 2 were positive for cytomegalovirus (CMV) on biopsy, complicating attribution solely to immunotherapy. Distinguishing between these entities is clinically challenging, as clinical presentation and endoscopic findings may overlap. The incidence of CMV reactivation among patients with solid tumors treated with ICIs is considered low, and CMV colitis is reported in about 3.8% of patients with relapsed or refractory immune-related colitis [26] and in 7.7% of patients treated with ICIs [27]. The identification of CMV-induced cytopathic changes on conventional hematoxylin and eosin (H&E)-stained tissue remains the gold standard for diagnosing CMV disease in patients with colitis, despite a highly variable sensitivity ranging from 10% to 87% [26]. IHC improves sensitivity to 78–93%, and therefore, the combination of H&E staining and IHC is highly recommended [28]. Polymerase chain reaction (PCR) techniques for the amplification of CMV DNA provide a diagnostic sensitivity from 92% to 96.7%, justifying the use of this method in our patients to enhance diagnostic accuracy, as failure to identify CMV involvement may lead to corticosteroid-refractory colitis or clinical deterioration [28].

The possible mechanisms for the CMV–colitis association in this setting are the following [23]: pure CMV colitis in immunosuppressed patients due to chemotherapy and/or corticosteroids [24]; CMV reactivation triggered by immune-mediated colitis [25]; or mixed immune-mediated colitis with CMV superinfection. As a precaution, CMV-positive patients were treated with VALGANCICLOVIR prior to corticosteroid initiation, with favorable outcomes. In fact, several reports have shown that CMV-positive patients benefit from antiviral therapy, initiated prior to or concomitantly with immunosuppressive treatment, resulting in symptom resolution and improved outcomes [29].

Finally, the observed association between grade 3–4 immune-related adverse events and higher pathological complete response rates should be interpreted with caution. Given the retrospective design, the limited sample size, and the absence of multivariable modeling, no causal relationship can be inferred. This observation should therefore be considered hypothesis-generating and warrants further investigation in larger, prospective cohorts.

The strengths of this study include a detailed description of immune- and chemotherapy-related toxicities by organ system, integrating clinical, biological, pathological, and radiological data. This multidimensional approach, resulting from collaboration between oncologists, clinical immunologists, and organ specialists, improves understanding of the expected toxicity profile and may assist clinicians in patient management. Our data show that the frequency of grades 3–4 toxicities is broadly comparable to that in KEYNOTE-522, and that these adverse events are, in most cases, manageable. Finally, we observed encouraging efficacy signals in our population, with a pCR rate comparable to that in KEYNOTE-522 (61.1% vs. 63%) [7] and a 100% overall survival rate at 30 months among patients achieving complete pathological response.

The limitations of this study include its retrospective design, which is subject to inherent biases (missing data, heterogeneity of reporting) and the potential underestimation of some clinical or laboratory toxicities due to the lack of systematic documentation.

Moreover, attributing certain toxicities specifically to immunotherapy or chemotherapy was sometimes challenging (e.g., diarrhea, transaminase elevation, troponin elevation), particularly for grade 1–2 events, where limited diagnostic work-up is usually performed—hence our focus on grade 3–4 toxicities for interpretation. Another limitation is the relatively short median follow-up (22 months), which precludes a robust analysis of recurrence-free survival and overall survival; these will be addressed in a subsequent publication after a longer follow-up.

## 5. Conclusions

- Grade 3–4 toxicities occurred in 20% of patients treated with neoadjuvant chemotherapy plus pembrolizumab for localized TNBC in our real-world practice.
- Patients with grade 3–4 irAEs tended to have higher pCR rates (80% vs. 56.8%; *p* = 0.079).
- The number of neoadjuvant pembrolizumab cycles did not influence the pCR rate.
- Approximately 16.3% of the cohort exhibited elevated troponin levels, and 2% had documented immune-related myocarditis.
- Postoperative troponin elevation could possibly be related to MINS; however, immune-related myocarditis should be ruled out first.
- Baseline ANA status does not appear to have predictive or prognostic value for severe toxicity occurrence.

These results are generally reassuring, especially as pembrolizumab is currently being investigated in other breast cancer subtypes, including hormone receptor-positive disease in the neoadjuvant setting, with promising pCR results from KEYNOTE-756 [30]. Survival data from this trial are forthcoming.

## Figures and Tables

**Table 1:** Epidemiological characteristics of the study population.

Age (Years)	
Median [range]	50 [27–76]
**Performance status (PS)**	
PS0	96.7%
PS1	3.3%
**Menopausal status**	
Menopausal	47.8%
Premenopausal	46.8%
Perimenopausal	5.4%
**History of hormonal intake**	
Yes	13%
No	29%
Unknown	58%
**Active smoking**	
Yes	34.4%
No	44.1%
Unknown	21.5%
**Alcohol intake**	
Yes	9.7%
No	44.1%
Unknown	46.2%
**BMI (kg/m^2^)**	
Median	24.17 [17–41.79]
**Comorbidities** *	
Yes	19.6%
No	80.4%
**History of autoimmune disease**	
Yes	3.3%
No	96.7%
**Personal history of breast cancer**	
Yes	7.6%
No	92.4%
**Pathogenic BRCA1/2 mutations**	
BRCA1	9.8%
BRCA2	1.1%

**BMI:** body mass index. * Comorbidities include hypertension, diabetes, dyslipidemia, renal insufficiency, history of ischemic disease (heart disease, stroke, and peripheral arterial disease), and thyroid dysfunction.

**Table 2:** Biological data and anti-immune antibodies at baseline.

LDH	
Elevated	7.7%
Normal	54.3%
Unknown	38%
**Albumin**	
Normal	65%
Unknown	35%
**Antinuclear antibodies (ANA)**	
Positive	17.4%
Negative	39.1%
Unknown	43.5%
**Rheumatoid factor**	
Positive	3.3%
Negative	47.8%
Unknown	48.9%
**Anti-CCP Ab**	
Positive	0.0%
Negative	45.7%
Unknown	54.3%
**Anti-TPO Ab**	
Positive	5.4%
Negative	45.7%
Unknown	48.9%

**LDH:** lactate dehydrogenase; **Anti-CCP Ab**: anti-cyclic citrullinated peptide antibodies; **anti-TPO** **Ab**: anti-thyroid peroxidase antibodies.

**Table 3:** Reasons for non-administration of pembrolizumab in the adjuvant setting (N = 32).

Reason for Non-Administration of Treatment	100% (N = 32)
Immunotherapy-related toxicity occurring during the neoadjuvant phase (irrespective of severity)	50%
Isolated troponin elevation on the pre-treatment workup for adjuvant therapy, with normal findings on cardiological investigations	18.8%
Disease progression	6.2%
Pathological complete response and decision not to pursue the adjuvant sequence	9.4%
Patient’s preference	12.5%
Anthracycline-related cardiac toxicity, with a preference to avoid adding immunotherapy in this context	3.1%

**Table 4:** Frequency of main toxicities observed during treatment sequences including pembrolizumab (neoadjuvant and adjuvant phases).

	PC-Pembrolizumab (N = 92)		EC-Pembrolizumab (N = 91)		Adjuvant Pembrolizumab (N = 60) ^a^	
Toxicity	Grades 1–2	Grades 3–4	Grades 1–2	Grades 3–4	Grades 1–2	Grades 3–4
Allergic reaction	18.5%	0	4.3%	0%	0%	0%
Skin toxicity *	23.9%	1.1%	9.8%	0	5%	1.7%
Diarrhea	38%	2.2%	8.7%	1.1%	3.3%	0%
Nausea	58.7	2.2%	52.2%	1.1%	0%	0%
Vomiting	16.3%	1.1%	17.4%	0%	0%	0%
Peripheral neuropathy	41.3%	1.1%	0%	0%	0% **	0%
Arthralgias	9.8%	0%	5.4%	0%	6%	1.6%
Myalgias	18.5%	0%	1.1%	0%	0%	0%
Mucitis	13%	0%	15.2%	1.1%	0%	0%
Constipation (de novo)	20.7%	0%	7.6%	0%	0%	0%
Thrombopenia	12%	1.1%	4.3%	1.1%	0%	0%
Anemia	58.7%	4.3%	53.3%	6.5%	0%	0%
Neutropenia	20.7%	43.5%	12%	18.5%	0%	0%
Febrile neutropenia	2.2%		17.4%		0%	0%
Fever	16.3%	12%	19.6	1.1%	0%	0%
Infections	22.8%	0%	14.1%	0%	5%	0%
Elevated ALT levels	16.5%	5%	4%	3%	15%	0%
Elevated Troponin levels	1%	0%	6.5%	0%	***	1.6%
Dysthyroïdism (de novo)	12%	0%	7.6%	1%	8.3%	0%
Adrenal insufficiency	3.2%	0%	2.1%	1%	3.3	0%
Renal toxicity	1%	0%	0%	0%	0%	0%
AIHA	0%	1%	0%	0%	0%	0%
Sicca syndrome	1%	0%	2.1%	0	1%	0%
Carpal tunnel syndrome	0%	0%	0%	0%	1%	0%

**PC:** paclitaxel–carboplatine. **EC:** epirubicine–cyclophosphamide. **ALT:** alanine aminotransferase. **AIHA:** autoimmune hemolytic anemia. **^a^**: Percentages are calculated based on the number of patients who received adjuvant pembrolizumab (N = 60). * Cutaneous toxicity does not include nail or hair toxicity. ** Pre-existing residual neuropathies were not taken into account; only de novo toxicities were recorded. *** Patients with troponin abnormalities during the pre-treatment workup for the adjuvant phase were not included; this issue will be addressed in the section dedicated to cardiac toxicity.

**Table 5:** Characteristics of observed cutaneous toxicities (N = 30; 100%).

Time to Onset (Weeks)	
Median	2.5 [1–88]
Mean	11.8
**Type of skin lesion**	
Isolated pruritus	26.7% (8% *)
Maculopapular exanthem (rash)	23.3% (7.7% *)
Isolated skin xerosis	6.7% (2% *)
Urticaria	16.7% (5.4% *)
Eczema	3.3% (1% *)
Acneiform eruption	3.3% (1% *)
Psoriasis	3.3% (1% *)
Disabling lichen	10% (3.2% *)
Eczema + Acneiform eruption	3.3% (1% *)
Suspicion of DRESS syndrome	3.3% (1% *)
**Clinical Grade (CTCAE v5.0)**	
Grades 1–2	93.3% (30.4% *)
Grades 3–4	6.7% (2.2% *)

* Percentage of the event relative to the total study population of 92 patients. **CTCAE v5.0**: Common Terminology Criteria for Adverse Events, version 5.0.

**Table 6:** Characteristics of immune-related endocrine toxicities (thyroid dysfunction and adrenal insufficiency).

	Thyroid Dysfunction N = 25; 100%	Adrenal Insufficiency N = 8; 100%
**Time to onset (weeks)**		
Mean	17 [4–44]	17.6 [8–33]
Median	14	13
**Timing of onset**		
Neoadjuvant	80% (21.7% of total cohort *)	75% (6/8)
Adjuvant	20% (5.4% of total cohort *)	25% (2/8)
**Severity (CTCAE v5.0)**		
Grades 1–2	95.7% (26% of total cohort *)	87.5% (7/8)
Grades 3–4	4.3% (1% of total cohort *)	12.5% (1/8)
**Anti-TPO antibodies**		
Positive	16%	-
Negative	32%	
Not tested	52%	
**Toxicity requiring hospitalization**	4%	12.5% (1/8)

**Anti-TPO Ab**: anti-thyroid peroxidase antibodies; **CTCAE v5.0**: Common Terminology Criteria for Adverse Events, version 5.0. * Total cohort refers to the whole study population (92 patients).

## Data Availability

Data are available from the corresponding author upon reasonable request.

## Supplementary Materials

The following supporting information can be downloaded at https://www.mdpi.com/article/10.3390/cancers18030358/s1. Table S1. Characteristics of Liver Toxicity Related to Treatment. Table S2. Characteristics of Liver Toxicity Related to Treatment.
