# Supplementary material for: Neoadjuvant Pembrolizumab Associated with Chemotherapy in Early Triple-Negative Breast Cancer Patients: Real-World Data from a French Single-Center Experience
Source: Cancers (Basel). 2026 Jan 23;18(3):358. doi: 10.3390/cancers18030358 (PMC12896850; doi:10.3390/cancers18030358)
Supplement: Supplementary file 1 [file cancers-18-00358-s001.zip › cancers-4055603-supplementary.pdf]

cancers

MDPI

Supplementary Materials:

Supplementary Table S1. Characteristics of Liver Toxicity Related to Treatment

N=33(=100%)	**Grade 1** (58%, N=19)	**Grade 2** (18% , N=6)	**Grade 3** (18%, N=6)	**Grade 4** (6%, N=2)
**Time to onset (weeks)**	15,8 [1-57]	5,5 [1-9]	6 [1-18]	20,5 [1-40]
**Presentation**				
**Cholestatic**	14/19	0/6	1/6	0/2
**Cytolytic**	4/19	0/6	4/6	0/2
**Mixed**	1/19	6/6	1/6	2/2
**Immune antibodies**				
**Negative**	5/19	4/6	5/6	2/2
**Positive**	3/19 (ANA*)	0/6	1/6 (ANA*)	0/2
**Not tested**	11/19	2/6	0/6	0/2
**Infectious serology tests**				
**Positive**	0/19	0/6	0/6	0/2
**Negative**	9/19	6/6	16/6	2/2
**Not tested**	10/19	0/6	0/6	0/2

**Biopsy**	No	No	1/6	2/2
**Corticosteroids**	0/19	Yes 1/6<br>No 5/6	Yes 1/6<br>No 5/6	Yes 2/2
**Delursan**	0/19	0/6	0/6	2/2
**Clinico-biological course**	Favorable outcome	Favorable outcome	Favorable outcome	Favorable outcome

**ANA (Anti-nuclear Antibodies) detected at a titer of 1:160 with a speckled pattern, but serum was negative for autoimmune hepatitis–specific antibodies.*

Supplementary Table S2. Characteristics of Liver Toxicity Related to Treatment

Patient	Clinical presentation	Endoscopic findings	Histology (including CMV assessment)	Management	Outcome
A	**58-year-old patient** with diarrhea grade 1–2 starting at week 3, progressing to grade 3 by week 10	Erythematous mucosa with bleeding on contact; no ulcerations	Inflammatory colitis consistent with immune-related etiology; CMV IHC negative; CMV PCR on biopsy positive (suggestive of viral superinfection); CMV serology: IgM negative, IgG positive; CMV PCR in blood negative	Valganciclovir followed by oral corticosteroids, then TNF inhibitor due to corticosteroid dependence	Clinical improvement
B	**44-year-old patient** with diarrhea starting at week 4, worsening to grade 3 by week 20	Severe proctitis and colitis with superficial ulcerations	Diffuse acute colitis possibly immune-related; CMV IHC negative; weakly positive CMV PCR on rectal biopsy;<br>CMV serology negative (IgG, IgM)	Valganciclovir for 15 days followed by oral corticosteroids	Clinical improvement
C	**46-year-old patient** with grade 3 diarrhea and rectal bleeding at week 3, followed by constipation	Normal rectum and colon; hemorrhoids and anal fissure	Moderate chronic interstitial follicular proctitis; CMV IHC negative	Symptomatic treatment only	Complete resolution
